# *catena*-Poly[[diaqua­lithium]-μ-[*rac-cis*-(2-carb­oxy­cyclo­hexane-1-carboxyl­ato-κ^2^ *O* ^1^:*O* ^2^]]

**DOI:** 10.1107/S1600536811045077

**Published:** 2011-11-05

**Authors:** Graham Smith, Urs D. Wermuth, Michael L. Williams

**Affiliations:** a Faculty of Science and Technology, Queensland University of Technology, GPO Box 2434, Brisbane, Queensland 4001, Australia; b School of Biomolecular and Physical Sciences, Griffith University, Nathan, 4111, Australia

## Abstract

In the structure of the title compound, [Li(C_8_H_11_O_4_)(H_2_O)_2_]_*n*_, the distorted tetra­hadral LiO_4_ coordination sphere comprises two water mol­ecules and two carboxyl O-atom donors from separate bridging *cis*-2-carb­oxy­cyclo­hexane-1-carboxyl­ate monoanions [Li—O = 1.887 (4)–1.946 (3) Å], giving chain substructures which extend along [010]. Water–water and water–carboxyl O—H⋯O hydrogen bonds stabilize these chain structures and provide inter­chain links, resulting in a two-dimensional layered structure extending parallel to (100).

## Related literature

For the structure of an Ni^II^ complex derived from racemic *cis*-cyclo­hexane-1,2-dicarb­oxy­lic acid, see: Zheng *et al.* (2008 ▶) and for the structure of the corresponding Sr^2+^ complex, see: Robertson & Harrison (2010 ▶). For the structure of lithium 3,5-dinitro­benzoate, see: Yang & Ng (2007 ▶) and for lithium hydrogenterephthalate pseudopolymorphs, see: Küppers (1978 ▶); Gonschorek & Küppers (1975 ▶); Adiwidjaja & Küppers (1978 ▶).
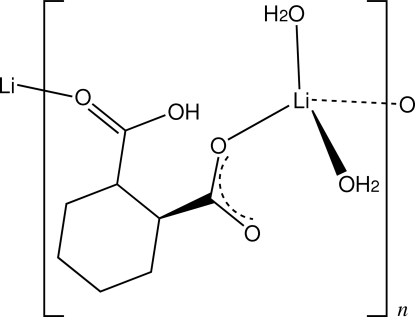


## Experimental

### 

#### Crystal data

[Li(C_8_H_11_O_4_)(H_2_O)_2_]
*M* *_r_* = 214.14
Monoclinic, 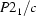

*a* = 16.2749 (5) Å
*b* = 5.4568 (2) Å
*c* = 12.0438 (5) Å
β = 97.533 (3)°
*V* = 1060.37 (7) Å^3^
*Z* = 4
Mo *K*α radiation
μ = 0.11 mm^−1^
*T* = 200 K
0.30 × 0.25 × 0.20 mm

#### Data collection

Oxford Diffraction Gemini-S CCD-detector diffractometer
Absorption correction: multi-scan (*CrysAlis PRO*; Oxford Diffraction, 2010 ▶) *T*_min_ = 0.97, *T*_max_ = 0.99
5758 measured reflections
2084 independent reflections
1550 reflections with *I* > 2σ(*I*)
*R*_int_ = 0.023

#### Refinement

*R*[*F* ^2^ > 2σ(*F* ^2^)] = 0.045
*wR*(*F* ^2^) = 0.134
*S* = 1.04
2084 reflections
136 parameters
H-atom parameters constrained
Δρ_max_ = 0.82 e Å^−3^
Δρ_min_ = −0.39 e Å^−3^

### 

Data collection: *CrysAlis PRO* (Oxford Diffraction, 2010 ▶); cell refinement: *CrysAlis PRO*; data reduction: *CrysAlis PRO*; program(s) used to solve structure: *SIR92* (Altomare *et al.*, 1994 ▶); program(s) used to refine structure: *SHELXL97* (Sheldrick, 2008 ▶) within *WinGX* (Farrugia, 1999 ▶); molecular graphics: *PLATON* (Spek, 2009 ▶); software used to prepare material for publication: *PLATON*.

## Supplementary Material

Crystal structure: contains datablock(s) global, I. DOI: 10.1107/S1600536811045077/ng5258sup1.cif

Structure factors: contains datablock(s) I. DOI: 10.1107/S1600536811045077/ng5258Isup2.hkl

Additional supplementary materials:  crystallographic information; 3D view; checkCIF report

## Figures and Tables

**Table 1:** Hydrogen-bond geometry (Å, °)

*D*—H⋯*A*	*D*—H	H⋯*A*	*D*⋯*A*	*D*—H⋯*A*
O1*W*—H11*W*⋯O11^i^	0.99	1.77	2.739 (2)	163
O1*W*—H12*W*⋯O1*W*^ii^	0.90	2.26	3.164 (3)	180
O2*W*—H21*W*⋯O12^iii^	0.90	1.89	2.7913 (19)	179
O2*W*—H22*W*⋯O11^iv^	0.87	2.13	2.936 (2)	153
O2*W*—H22*W*⋯O12^iv^	0.87	2.54	3.228 (2)	136
O22—H22⋯O11^v^	0.90	1.70	2.5958 (17)	174

Symmetry codes: (i) 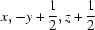; (ii) 
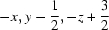
; (iii) 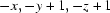; (iv) 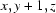; (v) 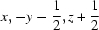.

**Fig. 1.**
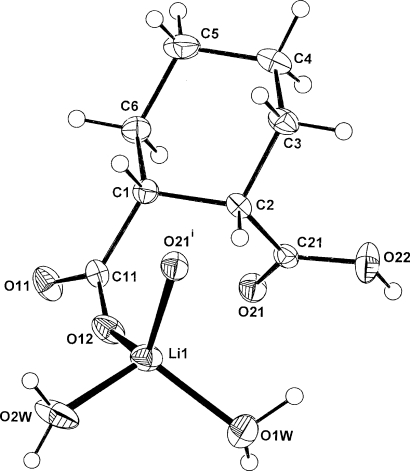
Molecular configuration and atom naming scheme for the complex unit in the title compound with displacement ellipsoids drawn at the 40% probability level. For symmetry code (i), see Table 1.

**Fig. 2.**
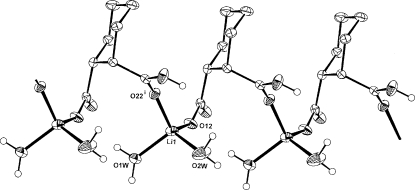
The hydrogen-bonded complex chain substructure of the title compound and its extension along [010]. Hydrogen bonds are shown as dashed lines and non-associative H atoms are omitted.

**Fig. 3.**
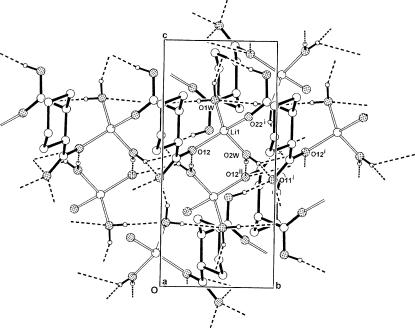
A view down the a axis of the unit cell showing the sheet structure extending parallel to (100). For symmetry codes, see Table 1.

## supplementary crystallographic information

### Comment

The structures of metal complexes with *cis*-cyclohexane-1,2-dicarboxylic acid (*cis*-CHDC) are not common in the crystallographic literature with examples largely involving metals of the first-row transition series, *e.g.* Ni^II^ (Zheng *et al.*, 2008), and also with Sr (Robertson & Harrison, 2010). In these complexes the *cis*-CHDC ligand is usually in the dianionic form. Our 1:1 stoichiometric reaction of *cis*-CHDC anhydride with lithium carbonate in 50% ethanol–water solution provided minor crystals of the title compound, [C_8_H_11_LiO_4_(H_2_O)_2_]_n_ in which the ligand is in the monoanionic form and the structure is reported here.

In this complex (Fig. 1), the LiO_4_ stereochemistry is distorted tetrahedral, involving two water molecules and two O-donors from separate carboxyl groups of the monoanions in a bridging mode [Li—O range, 1.887 (4)–1.946 (3) Å]. This coordination mode is usual for Li carboxylate complexes, *e.g.*, the analogous pseudopolymorphs of lithium hydrogen phthalate (the monohydrate, the dihydrate and the methanol monosolvate: Gonschorek & Küppers, 1975; Küppers, 1978; Adiwidjaja & Küppers, 1978) and with lithium 3,5-dinitrobenzoate (Yang & Ng, 2007). In the title compound, the complex units form one-dimensional chain substructures which extend along [010] (Fig. 2). Within the chains, water–water and water–carboxyl hydrogen bonds, including a three-centre *O*—*H*···O_carboxyl_ cyclic interaction involving O2*W* and infinite water chains involving O1*W* (Table 1), stabilize the structure. In addition, a strong carboxylic acid–carboxyl O—H···O hydrogen bond and water–carboxyl hydrogen bonds from both water donors link the chains, resulting in a two-dimensional layered structure extending parallel to (100) (Fig. 3).

### Experimental

The title compound was synthesized by heating under reflux for 10 min. a solution of 1 mmol of cyclohexane-1,2-dicarboxylic anhydride and 1 mmol of lithium carbonate in 50 ml of 1:1 ethanol–water. After concentration to *ca* 30 ml the filtered solution was allowed to evaporate at room temperature, giving finally a residual viscous oil in which minor well-formed colourless crystals of the title compound were found.

### Refinement

Hydrogen atoms involved in hydrogen-bonding interactions were located by difference-Fourier methods and their positional parameters were allowed to ride with *U*_iso_(H) = 1.5*U*_eq_(O). Other hydrogen atoms were included in the refinement at calculated positions [C—H = 0.97–0.98 Å] with *U*_iso_(H) = 1.5*U*_eq_(C)]. The residual positive electron density in the refinement (0.823 e Å^-3^) is unusually large for this structure and is located 1.02 Å from H12*W*.

### Crystal data

**Table:**

[Li(C_8_H_11_O_4_)(H_2_O)_2_]	*F*(000) = 456
*M**_r_* = 214.14	*D*_x_ = 1.341 Mg m^−^^3^
Monoclinic, *P*2_1_/*c*	Mo *K*α radiation, λ = 0.71073 Å
Hall symbol: -P 2ybc	Cell parameters from 2836 reflections
*a* = 16.2749 (5) Å	θ = 3.2–28.7°
*b* = 5.4568 (2) Å	µ = 0.11 mm^−^^1^
*c* = 12.0438 (5) Å	*T* = 200 K
β = 97.533 (3)°	Block, colourless
*V* = 1060.37 (7) Å^3^	0.30 × 0.25 × 0.20 mm
*Z* = 4	

### Data collection

**Table:**

Oxford Diffraction Gemini-S CCD-detector diffractometer	2084 independent reflections
Radiation source: Enhance (Mo) X-ray source	1550 reflections with *I* > 2σ(*I*)
graphite	*R*_int_ = 0.023
Detector resolution: 16.077 pixels mm^-1^	θ_max_ = 26.0°, θ_min_ = 3.4°
ω scans	*h* = −19→20
Absorption correction: multi-scan (*CrysAlis PRO*; Oxford Diffraction, 2010)	*k* = −6→6
*T*_min_ = 0.97, *T*_max_ = 0.99	*l* = −13→14
5758 measured reflections	

### Refinement

**Table:**

Refinement on *F*^2^	Primary atom site location: structure-invariant direct methods
Least-squares matrix: full	Secondary atom site location: difference Fourier map
*R*[*F*^2^ > 2σ(*F*^2^)] = 0.045	Hydrogen site location: inferred from neighbouring sites
*wR*(*F*^2^) = 0.134	H-atom parameters constrained
*S* = 1.04	*w* = 1/[σ^2^(*F*_o_^2^) + (0.085*P*)^2^] where *P* = (*F*_o_^2^ + 2*F*_c_^2^)/3
2084 reflections	(Δ/σ)_max_ < 0.001
136 parameters	Δρ_max_ = 0.82 e Å^−^^3^
0 restraints	Δρ_min_ = −0.39 e Å^−^^3^

### Special details

**Table:**

Geometry. Bond distances, angles *etc*. have been calculated using the rounded fractional coordinates. All su's are estimated from the variances of the (full) variance-covariance matrix. The cell e.s.d.'s are taken into account in the estimation of distances, angles and torsion angles
Refinement. Refinement of *F*^2^ against ALL reflections. The weighted *R*-factor *wR* and goodness of fit *S* are based on *F*^2^, conventional *R*-factors *R* are based on *F*, with *F* set to zero for negative *F*^2^. The threshold expression of *F*^2^ > σ(*F*^2^) is used only for calculating *R*-factors(gt) *etc*. and is not relevant to the choice of reflections for refinement. *R*-factors based on *F*^2^ are statistically about twice as large as those based on *F*, and *R*- factors based on ALL data will be even larger.

### Fractional atomic coordinates and isotropic or equivalent isotropic displacement parameters (Å^2^)

**Table:**

	*x*	*y*	*z*	*U*_iso_*/*U*_eq_	
O1W	0.04966 (10)	0.4570 (4)	0.75941 (13)	0.0617 (7)	
O2W	0.03531 (8)	0.7437 (3)	0.53796 (16)	0.0527 (6)	
O11	0.17016 (8)	−0.0191 (2)	0.43846 (11)	0.0318 (5)	
O12	0.13158 (8)	0.2623 (2)	0.55259 (11)	0.0297 (4)	
O21	0.19747 (8)	−0.2424 (2)	0.69312 (11)	0.0265 (4)	
O22	0.23639 (9)	−0.0915 (2)	0.86385 (11)	0.0366 (5)	
C1	0.27299 (10)	0.1392 (3)	0.58486 (14)	0.0208 (5)	
C2	0.27190 (10)	0.1422 (3)	0.71265 (14)	0.0198 (5)	
C3	0.36052 (11)	0.1758 (3)	0.77421 (16)	0.0270 (6)	
C4	0.41960 (11)	−0.0170 (4)	0.73895 (17)	0.0331 (6)	
C5	0.42078 (12)	−0.0142 (4)	0.61278 (18)	0.0362 (7)	
C6	0.33353 (11)	−0.0509 (3)	0.54969 (16)	0.0265 (6)	
C11	0.18554 (11)	0.1254 (3)	0.52073 (14)	0.0211 (5)	
C21	0.23163 (10)	−0.0844 (3)	0.75461 (14)	0.0204 (5)	
Li1	0.10696 (18)	0.5409 (6)	0.6350 (3)	0.0278 (9)	
H1	0.29500	0.29900	0.56620	0.0310*	
H2	0.23920	0.28440	0.73030	0.0300*	
H11W	0.09160	0.44600	0.82710	0.0930*	
H12W	0.02120	0.31460	0.75400	0.0930*	
H21W	−0.01870	0.74220	0.50990	0.0790*	
H22	0.21350	−0.23120	0.88480	0.0550*	
H22W	0.06250	0.85130	0.50340	0.0790*	
H31	0.38070	0.33750	0.75810	0.0410*	
H32	0.35930	0.16460	0.85440	0.0410*	
H41	0.47500	0.01400	0.77660	0.0500*	
H42	0.40260	−0.17780	0.76160	0.0500*	
H51	0.45690	−0.14350	0.59240	0.0540*	
H52	0.44290	0.14110	0.59100	0.0540*	
H61	0.33560	−0.03770	0.46980	0.0400*	
H62	0.31400	−0.21400	0.56480	0.0400*	

### Atomic displacement parameters (Å^2^)

**Table:**

	*U*^11^	*U*^22^	*U*^33^	*U*^12^	*U*^13^	*U*^23^
O1W	0.0409 (9)	0.1113 (15)	0.0325 (9)	−0.0244 (10)	0.0032 (7)	0.0115 (9)
O2W	0.0242 (8)	0.0398 (9)	0.0898 (14)	−0.0014 (7)	−0.0090 (7)	0.0226 (9)
O11	0.0366 (8)	0.0306 (8)	0.0258 (8)	0.0077 (6)	−0.0050 (6)	−0.0094 (6)
O12	0.0245 (7)	0.0301 (7)	0.0325 (8)	0.0082 (6)	−0.0034 (5)	−0.0086 (6)
O21	0.0291 (7)	0.0253 (7)	0.0244 (7)	−0.0085 (6)	0.0005 (5)	−0.0021 (5)
O22	0.0573 (10)	0.0320 (8)	0.0197 (8)	−0.0171 (7)	0.0016 (6)	0.0011 (6)
C1	0.0213 (9)	0.0184 (9)	0.0227 (10)	−0.0009 (8)	0.0029 (7)	0.0018 (7)
C2	0.0199 (9)	0.0170 (9)	0.0214 (9)	0.0011 (7)	−0.0018 (7)	−0.0017 (7)
C3	0.0224 (9)	0.0258 (10)	0.0309 (11)	−0.0058 (8)	−0.0038 (7)	−0.0010 (8)
C4	0.0187 (9)	0.0363 (11)	0.0420 (13)	0.0012 (9)	−0.0042 (8)	0.0001 (9)
C5	0.0211 (10)	0.0431 (13)	0.0451 (13)	0.0046 (9)	0.0071 (8)	−0.0002 (10)
C6	0.0239 (9)	0.0295 (10)	0.0268 (10)	0.0048 (8)	0.0061 (7)	−0.0006 (8)
C11	0.0250 (9)	0.0194 (9)	0.0180 (9)	0.0014 (8)	−0.0004 (7)	0.0010 (7)
C21	0.0184 (8)	0.0210 (9)	0.0210 (9)	0.0039 (7)	0.0000 (7)	0.0004 (7)
Li1	0.0223 (15)	0.0305 (17)	0.0303 (17)	−0.0018 (14)	0.0029 (12)	−0.0068 (13)

### Geometric parameters (Å, °)

**Table:**

O1W—Li1	1.921 (4)	C2—C3	1.544 (2)
O2W—Li1	1.896 (4)	C2—C21	1.517 (2)
O12—Li1	1.887 (4)	C3—C4	1.523 (3)
O21—Li1^i^	1.946 (3)	C4—C5	1.522 (3)
O11—C11	1.265 (2)	C5—C6	1.533 (3)
O12—C11	1.251 (2)	C1—H1	0.9800
O21—C21	1.222 (2)	C2—H2	0.9800
O22—C21	1.308 (2)	C3—H31	0.9700
O1W—H11W	0.9900	C3—H32	0.9700
O1W—H12W	0.9000	C4—H41	0.9700
O2W—H21W	0.9000	C4—H42	0.9700
O2W—H22W	0.8700	C5—H51	0.9700
O22—H22	0.9000	C5—H52	0.9700
C1—C2	1.542 (2)	C6—H61	0.9700
C1—C11	1.530 (2)	C6—H62	0.9700
C1—C6	1.528 (2)		
			
C11—O12—Li1	148.04 (15)	C1—C2—H2	108.00
C21—O21—Li1^i^	155.44 (16)	C21—C2—H2	108.00
Li1—O1W—H11W	108.00	C2—C3—H32	109.00
Li1—O1W—H12W	116.00	C2—C3—H31	109.00
H11W—O1W—H12W	107.00	C4—C3—H32	109.00
Li1—O2W—H21W	136.00	H31—C3—H32	108.00
Li1—O2W—H22W	112.00	C4—C3—H31	109.00
H21W—O2W—H22W	111.00	C3—C4—H41	109.00
C21—O22—H22	110.00	C5—C4—H41	109.00
C2—C1—C6	112.09 (14)	C5—C4—H42	109.00
C2—C1—C11	111.90 (13)	C3—C4—H42	109.00
C6—C1—C11	114.69 (14)	H41—C4—H42	108.00
C1—C2—C3	110.39 (14)	C6—C5—H52	109.00
C1—C2—C21	112.71 (14)	H51—C5—H52	108.00
C3—C2—C21	110.74 (14)	C6—C5—H51	109.00
C2—C3—C4	111.61 (15)	C4—C5—H51	109.00
C3—C4—C5	111.25 (17)	C4—C5—H52	109.00
C4—C5—C6	111.18 (16)	C1—C6—H61	109.00
C1—C6—C5	111.27 (15)	C1—C6—H62	109.00
O12—C11—C1	117.38 (15)	C5—C6—H61	109.00
O11—C11—O12	122.52 (16)	H61—C6—H62	108.00
O11—C11—C1	120.10 (15)	C5—C6—H62	109.00
O21—C21—C2	123.74 (15)	O1W—Li1—O12	112.12 (18)
O21—C21—O22	123.43 (15)	O1W—Li1—O21^ii^	106.63 (18)
O22—C21—C2	112.83 (14)	O2W—Li1—O21^ii^	104.03 (17)
C11—C1—H1	106.00	O12—Li1—O21^ii^	118.41 (16)
C6—C1—H1	106.00	O2W—Li1—O12	107.57 (19)
C2—C1—H1	106.00	O1W—Li1—O2W	107.32 (16)
C3—C2—H2	108.00		
			
Li1—O12—C11—O11	−161.8 (3)	C11—C1—C6—C5	176.49 (15)
Li1—O12—C11—C1	17.7 (4)	C2—C1—C11—O11	−135.69 (16)
C11—O12—Li1—O1W	−115.3 (3)	C2—C1—C11—O12	44.8 (2)
C11—O12—Li1—O2W	126.9 (3)	C6—C1—C11—O11	−6.6 (2)
C11—O12—Li1—O21^ii^	9.5 (4)	C6—C1—C11—O12	173.93 (15)
Li1^i^—O21—C21—O22	51.7 (4)	C1—C2—C3—C4	−54.63 (19)
Li1^i^—O21—C21—C2	−127.4 (3)	C21—C2—C3—C4	70.90 (19)
C21—O21—Li1^i^—O1W^i^	−32.5 (4)	C1—C2—C21—O21	−5.8 (2)
C21—O21—Li1^i^—O2W^i^	80.8 (4)	C1—C2—C21—O22	174.98 (14)
C21—O21—Li1^i^—O12^i^	−159.9 (3)	C3—C2—C21—O21	−130.05 (17)
C6—C1—C2—C3	53.79 (18)	C3—C2—C21—O22	50.76 (19)
C6—C1—C2—C21	−70.61 (18)	C2—C3—C4—C5	56.5 (2)
C11—C1—C2—C3	−175.75 (13)	C3—C4—C5—C6	−56.5 (2)
C11—C1—C2—C21	59.85 (18)	C4—C5—C6—C1	55.4 (2)
C2—C1—C6—C5	−54.49 (19)		

Symmetry codes: (i) *x*, *y*−1, *z*; (ii) *x*, *y*+1, *z*.

### Hydrogen-bond geometry (Å, °)

**Table:**

*D*—H···*A*	*D*—H	H···*A*	*D*···*A*	*D*—H···*A*
O1W—H11W···O11^iii^	0.99	1.77	2.739 (2)	163
O1W—H12W···O1W^iv^	0.90	2.26	3.164 (3)	180
O2W—H21W···O12^v^	0.90	1.89	2.7913 (19)	179
O2W—H22W···O11^ii^	0.87	2.13	2.936 (2)	153
O2W—H22W···O12^ii^	0.87	2.54	3.228 (2)	136
O22—H22···O11^vi^	0.90	1.70	2.5958 (17)	174
C3—H32···O22	0.97	2.45	2.819 (2)	102

Symmetry codes: (iii) *x*, −*y*+1/2, *z*+1/2; (iv) −*x*, *y*−1/2, −*z*+3/2; (v) −*x*, −*y*+1, −*z*+1; (ii) *x*, *y*+1, *z*; (vi) *x*, −*y*−1/2, *z*+1/2.
